# Supplementary material for: Ultrasonic flexural mode emitters: New approaches to increasing sound pressure during oscillation generation in gas media
Source: PLoS One. 2025 Nov 21;20(11):e0336776. doi: 10.1371/journal.pone.0336776 (PMC12637920; doi:10.1371/journal.pone.0336776)
Supplement: Attachment — Submitted filename: Answers.docx [file pone.0336776.s006.docx]

**Reviewer 1**

**Thank you very much for your valuable suggestions regarding our work. Below we have tried to provide answers to all questions. Appropriate corrections have been made to the article. We hope that after the correction the article became significantly better.**

1. The manuscript should be structured properly, and headings should be numbered.

**Thank you for your comment. We have made every effort to align the manuscript's structure with PLOS ONE journal requirements. We have numbered the headings. The corresponding corrections have been made to the text.**

2. It is suggested to cite more recent literature (2020–2025), especially from outside the authors' own group.

**Thank you for your comment. The corresponding corrections have been made to the list of references. Currently at least 38% of the total number are sources from 2020-2025.**

3. In the introduction section, provide the background of the topic, and motivation and objectives of this work. Summarize the related work in literature and narrow down to the research gap. Also include a comparative table for modern ultrasonic emitters.

**Thank you. We completely agree with you. We have completely rewritten the introduction to provide basic information about the research topic and the current state of the art. We also outline the current research challenges and our proposed solutions. A comparative table of ultrasonic emitter characteristics has been added at the end of the introduction (Table 1).**

4. The authors have reported SPL increases, however, uncertainty ranges, error bars, and number of repetitions are missing in the results.

**Thank you for noticing this unfortunate oversight. We've updated the article with more detailed information about the experimental setup (page 14), uncertainty ranges for the SPL values (page 15), and information on the number of repeated measurements (page 15).**

Unfortunately, adding error bars to the graphs overcrowds them with overlapping bars and thus makes them unreadable. We have added the description of the error ranges regarding measurements in the text of the article (see page 15)

5. The manuscript has several grammatical errors. Please consider thorough language editing.

**Thank you for bringing this error to our attention. We've reviewed the article and attempted to correct any spelling errors.**

6. Pleaseincludeanomenclatureto define all symbols along with their units.

**Thank you for your comment. A list of all symbolic notations and units of measurement has been added to the article as Appendix 1.**

7. Please ensure that all the data is provided with the supporting files.

**Thank you for your comment. We've attached the data needed to construct the radiation patterns to the article.**

**Reviewer 2:**

**Thank you very much for your time and appreciation of our manuscript. We tried to take into account all your suggestions and correct your comments. We hope that after correction the manuscript has become significantly better.**

1. Abstract. The abstract also should present a brief topic framework, describe the scope and objectives, in addition to present the methodology adopted ind the main results/conclusions.

**Thank you for your work. The abstract has now been updated to include the purpose of the research, allowing for a clearer understanding of the article's focus. The abstract presents the main results achieved during the study in more detail. Potential applications of the research findings are outlined, particularly the generation of directional acoustic fields for solving a wide range of technological problems.**

2. Page 8, paragraph 2. Replace “wave,and” with “wave, and”.

**Thank you for noticing this unfortunate oversight. We'vecorrectedit.**

3. Page 10. Paragraph 1 after Fig. 5. variables should be italicised.

**Thank you. After Figure 5, the variables are highlighted in italics.**

4. Table 2. Overall dimensions, mm: disc diameter d x D. Could authors clarify the meaning of "x D"?, For example, in disc diameter 146 mm, it is 146x200.

**Thank you for your comment. The second 200 value (in the example provided), is the length of the emitter assembly with the piezoelectric transducer (the linear dimension along the centerline of the disk emitter). Since the disk diameter is known from the table column headers, we have changed the table format. This row now only indicates the emitter length.**

5. Figures 9, 10, 12, 16. All graphs could have the same scale to make it easier to compare the results. For example, 110, 115, 120,... 160.

**Thank you for your attention. The graphs in Figures 9, 10, 12, and 16 are shown to the same scale.**

6. Figures 9, 10, 12, 16. The units should be shown.

**Thank you. We have indicated the units of measurement in figures 9, 10, 12, and 16.**

**Reviewer №3**:

**Thank you very much for the work done and the critical comments provided on the article in question.**

Anyway, the goal of the work must be better illustrated within the abstract, introduction and conclusions. Compared with other research articles, which treat the same topic, the superiority of the manuscript has not been clearly explained. So, please review the corresponding parts. Moreover, please make sure that all the software, which have been utilized, have been referenced. The further work should additionally be mentioned at the end of the article. Furthermore, some sentences within the text are too long. So, spelling, sentence structure, conciseness and writing style could be improved with the help of a native English speaker. In conclusion, an “appendix” section, containing names and elaboration of the symbols and acronyms used, should also be inserted at the end of the article.

**Thank you. We have added the abstract which describes the purpose of the research in more detail (p. 2). The introduction presents a breakdown of the stated goal for achieving it through solving the stated objectives (p. 3).**

**We carefully read and edited the text with the help of a native English speaker, making every effort to remove long sentences and correct spelling.**

**Links to the software used in the research are provided on page 15.**

**The Conclusions section (pp. 21, 22) presents the solved objectives, providing qualitative and quantitative data for each. The conclusion also highlights future prospects that arise from continuing the research topic.**

**The article is supplemented with an Appendix A containing a detailed description of the symbols and abbreviations used.**
